# Real-World Outcomes of a Digital Behavioral Coaching Intervention to Improve Employee Health Status: Retrospective Observational Study

**DOI:** 10.2196/50356

**Published:** 2024-09-10

**Authors:** Amani Fadzlina Abdul Aziz, Tiffanie Ong

**Affiliations:** 1 Naluri Hidup Sdn Bhd Kuala Lumpur Malaysia

**Keywords:** digital behavioral coaching, chronic disease management, digital health, mHealth, workplace interventions, mobile phone

## Abstract

**Background:**

Chronic noncommunicable diseases (NCDs) account for major disability and premature mortality worldwide, with low- and middle-income countries being disproportionately burdened. Given the negative impact of NCDs on employee performance and work productivity, there is a rising need for stakeholders to identify effective workplace solutions that can improve employee health outcomes. As the workplace becomes more dispersed post pandemic, digital behavioral coaching offers a scalable, personalized, and cost-effective method of managing chronic disease risk factors among employees.

**Objective:**

This study aimed to retrospectively evaluate the impact of a digital behavioral coaching program on year-to-year changes in employee health status in a cohort of Indonesian employees.

**Methods:**

This retrospective real-world exploratory analysis of secondary health data followed 774 employees of an Indonesian company who completed company-sponsored health screenings between 2021 and 2022 and were given access to Naluri (Naluri Hidup Sdn Bhd), a holistic digital therapeutics platform offering digital behavioral health coaching and self-help tools. Participants were retrospectively classified as those who received active coaching (n=177), passive coaching (n=108), and no coaching (n=489). Linear mixed-effects models were used to evaluate the year-to-year changes in health outcomes across the 3 employee groups, with post hoc analyses evaluating within-group differences between the 2 time points and between-group differences at follow-up.

**Results:**

Significant time×group interaction effects were detected for body weight, BMI, hemoglobin A_1c_, low-density lipoprotein, total cholesterol, and systolic and diastolic blood pressure. Post hoc pairwise comparisons revealed significant improvements in hemoglobin A_1c_ (mean difference [M_diff_]=–0.14, *P*=.008), high-density lipoprotein (M_diff_=+2.14, *P*<.001), and total cholesterol (M_diff_=–11.45, *P*<.001) for employees in the Active Coaching group between 2021 and 2022, with the other 2 groups reporting deteriorations in multiple health outcomes throughout the 2 time points. At follow-up, those who received active coaching between 2021 and 2022 reported significantly lower body weight (*P*<.001), BMI (*P*=.001), low-density lipoprotein (*P*=.045), and total cholesterol (*P*<.001) than the No Coaching group.

**Conclusions:**

This study demonstrates real-world outcomes and implications supporting the use of workplace digital behavioral coaching in improving employee health status. Given the rising burden of NCDs in the Southeast Asian region, our findings underscore the role that workplace digital health interventions can play in preventing and managing chronic disease risk factors.

## Introduction

Chronic noncommunicable diseases (NCDs) are responsible for 74% of deaths worldwide [1], and approximately 38% (15.2 million) of these deaths occur prematurely, affecting people aged 30 to 70 years [2]. The World Health Organization (WHO) estimates that 69% of all deaths in the Southeast Asian region are due to NCDs [3], which roughly translates to 9 million of the regional population dying each year from NCDs, with 52% of deaths occurring prematurely in those younger than 70 years of age [3]. In Indonesia, NCDs are estimated to account for 76% of all deaths in the country [1], with cardiovascular diseases and diabetes accounting for an estimated 35% and 6% of proportional mortality, respectively [4]. Indonesia’s rapid urbanization over the past decade has not only led to higher individual life expectancy but also higher rates of modifiable NCD risk factors such as unhealthy diet choices and sedentary lifestyles, ultimately contributing to the rise of an aging population at risk of developing NCDs [5].

Largely due to stretched and overburdened health care systems, low- and middle-income countries are disproportionately affected by the burden of NCDs [6]. The COVID-19 pandemic has also disrupted essential health service provision for NCDs in the region, further burdening health care systems and creating long-term impacts on health and mortality in the region [7]. Similarly, although Indonesia has been shifting its health care disease burden from communicable diseases to NCDs [5], the COVID-19 pandemic has largely disrupted the nation’s NCDs services in primary health care [8], highlighting the need for a multi-stakeholder, multi-component, and preventative approach to managing NCDs in the nation. As health care systems worldwide continue to tackle the downstream impact of the COVID-19 pandemic, a growing body of evidence sheds light on the potential of digital health as a resource-efficient solution for health care systems and health care payers [9,10], making their real-world uptake more relevant for NCD prevention as traditional health care systems in the region continue to be overburdened.

Beyond health care, workplace-level interventions have a high magnitude of impact in addressing and managing chronic disease risk factors [11]. Working adults spend more than a third of their lives in the workplace [12], which makes them increasingly vulnerable to work-related risk factors such as sedentary work, work stress, and easy access to unhealthy food [13]. For this reason, the WHO recommends workplace health promotion programs as an effective avenue to target physical inactivity and unhealthy dietary habits—two important modifiable risk factors for obesity, hypertension, diabetes, and hypercholesterolemia [14]. Although workplace health improvement initiatives have traditionally been physical and face-to-face in nature, the COVID-19 pandemic has highlighted the need for digital offerings that provide more accessible, personalized, and diverse offerings, that are becoming increasingly more relevant as offices become more dispersed in a post–COVID-19 world. Given that unmanaged NCDs adversely affect employers through increased health care costs, rising premiums, and increased work absenteeism and presenteeism [15,16], there is a rising need for employers to effectively use the workplace as an avenue for health promotion and NCD prevention.

Major modifiable risk factors for NCDs are often behavioral in nature, such as tobacco use, excessive alcohol use, unhealthy diets, and physical inactivity [17]. For this reason, interventions that aim to prevent or self-manage chronic disease risk factors often rely on behavioral change frameworks to create long-term and sustainable behavioral changes [18-20]. Digital behavioral health coaching is a scalable and accessible method of delivering patient-centered health coaching, which is defined by an individual’s interpersonal relationship with a certified coach that allows individuals to develop intrinsic motivation, create sustainable change, and increase accountability through the use of behavioral change theory, motivational strategies, and communication techniques [21]. Concurrently, there is growing evidence to support the effectiveness of digital workplace behavioral coaching interventions in improving various chronic disease risk factors [22], such as weight and BMI [23-27], waistline circumference [26,28], blood pressure (BP) [23,26,28], cholesterol levels [26,28], and blood glucose levels [28].

A majority of studies evaluating digital workplace interventions are based in Western or high-income countries. Different health system capacities, income levels, health literacy levels, and health-seeking cultures necessitate more studies that are localized within the region to more accurately evaluate the impact of digital workplace interventions in the Southeast Asian population [29]. This study leverages a retrospective analysis of secondary employee health data to fill in the current gap in digital workplace health interventions in the region, by exploring year-to-year changes in employee physical health status associated with different levels of coaching delivered by Naluri, a Southeast Asian digital therapeutics platform, as part of a workplace health improvement program implemented in an Indonesian mining company. We focus on changes in BMI, waistline circumference, lipoprotein and total cholesterol levels, hemoglobin A_1c_ (HbA_1c_), BP—all of which are primary indicators of obesity, hypercholesterolemia, diabetes, and hypertension, respectively.

## Methods

### Study Design

This was a retrospective, observational, and exploratory study using secondary, longitudinal data from a cohort of employees at a prominent mining company in Indonesia.

### Settings and Participants

Indonesian labor regulations stipulate that employers conduct regular mandatory health examinations to assess the physical health and ability of employees [30]. In 2021 and 2022, employees of a mining company were invited to complete a company-sponsored health screening that measured current employee health profiles such as weight, BP, blood glucose, and cholesterol levels. Participants of this study were those who had completed the annual company-sponsored health screening in 2021 and 2022, with the inclusion criteria set to employees with valid and matching health data across the 2 health screenings. Exclusion criteria were set to (1) employees with missing health data, who did not complete either one or both health screenings in 2021 and 2022, (2) employees reporting an active pregnancy status during either or both health screenings in 2021 and 2022, and (3) employees whose health data were considered as unreasonable or improbable due to extreme outliers beyond clinically realistic levels.

After completing their health screening in 2021, employees were given access to receive digital health services provided by Naluri as part of their employer-provided employee wellness benefits. Employees were given the autonomy and freedom to voluntarily receive Naluri’s digital health services, with no external coercion or pressure to join from their employers or from Naluri personnel. Based on the employee health profile from the 2021 health screening, 300 employees with the highest risk factor indicators for obesity, hypercholesterolemia, diabetes, and hypertension were then identified and invited to join a 16-week structured, intensive digital chronic disease management program (CDMP) delivered on the Naluri mobile app (Naluri Hidup Sdn Bhd) at 2 points throughout the year, that is, in April 2022, and later in June 2022. Employees were invited to join Naluri’s CDMP if their 2021 health screening outcomes met 2 or more of the following criteria: (1) HbA_1c_≥5.7%; (2) total cholesterol level≥201.08 mg/dL; (3) BMI≥23 kg/m^2^; or (4) systolic BP≥120 mm Hg and diastolic BP≥80 mm Hg. At the end of 2022, all employees, regardless of use or engagement with Naluri services, were again invited by their employers to participate in an annual company-sponsored health screening.

Employees who fulfilled this study’s inclusion criteria were retrospectively categorized into three groups, which were (1) Active Coaching group, comprising employees who were invited and voluntarily enrolled into a 16-week, active, coach-led structured intensive digital CDMP during the interval between the 2021 and 2022 health screening; (2) Passive Coaching group, comprising of employees who voluntarily accessed self-led, unstructured, and passive digital behavioral coaching on the Naluri app through their employee assistance program (EAP) during the interval between the 2021 and 2022 health screening; and (3) No Coaching group, which are employees who reported no use of Naluri digital health services during the interval between the 2021 and 2022 health screening.

### Intervention

#### Active Coaching: Naluri CDMP

The Naluri CDMP is a 16-week intensive disease prevention program that uses a multidisciplinary team–based approach to improve physical health outcomes and chronic disease risk factors, primarily targeting indicators for obesity, hypercholesterolemia, diabetes, and hypertension. Naluri adopts the transtheoretical model of behavioral change [31] to help individuals adopt and maintain lifestyle habits that can sustainably improve health outcomes. The 16-week CDMP comprises 5 stages. First, Getting Started, where individuals get onboarded onto the program, build rapport with their assigned coaches, and set baselines and expectations. Second, Preparation, where individuals discover their current needs and motivation, discuss habit formation and planning, and set behavioral goals. Third, Action, which involves skill building across 3 domains—behavior, cognitive, and emotion regulation—to help individuals build healthy habits, adjust mindsets, and manage emotions. Fourth, Resilience, which focuses on empowering individuals to overcome challenges, with emphasis on resilience boosting modules such as dealing with relapses, setbacks, and weight plateaus. Finally, Maintaining Success, which focuses on maintenance and inspiring accountability to prepare individuals for long-term, sustainable, and independent healthy living. Throughout each stage of the program, participants had access to a multidisciplinary team of health coaches, such as dietitian, fitness coach, medical advisors, and more, led by a mental health coach (master’s-level health or clinical psychologist or a certified counsellor), who follow a comprehensive curriculum rooted in cognitive behavioral therapy and motivational interviewing to provide dedicated and continuous holistic support to help goal setting and habit formation.

Each week of the CDMP starts with a weekly check-in with a respective coach (eg, psychologist, dietitian, fitness coach, or medical advisor), where each coach engages in individual text-based coaching over the Naluri mobile app. Coaches were provided a coaching framework to follow throughout the program, with coaching objectives set for each week of the CDMP. For example, in week 2, which falls under the “Preparation” stage, mental health coaches were tasked to assess for participants’ current mindsets, behaviors, barriers, and health goals, dietitians were tasked to assess for past dietary attempts for weight management and current eating patterns, while fitness coaches were tasked to assess for participants’ current and past exercise history. In addition, coaches were also tasked to assign participants to complete educational modules to supplement the objectives and goals for each week of the program. Throughout the 16 weeks, participants were also assigned to complete and update their thought, food, weight, and exercise journals, at a minimum of once per week. Although the CDMP involved a structured curriculum and framework, the coaching received by the participants were also personalized to tailor to their current health needs and goals.

#### Passive Coaching: Naluri EAP

Naluri’s EAP is a passive, self-led, and digitally-delivered service for employees seeking mental or physical health support. Although the Naluri EAP provides individuals with access to holistic digital health coaching from a multidisciplinary team of health care professionals, unlike the CDMP, the Naluri EAP is an unstructured and low-intensive service that allows individuals to personalize their own health improvement journey in a nonlinear fashion. The Active Coaching group received structured and proactive coaching by the CDMP whereas those in the Passive Coaching group were free to use any of Naluri’s digital services as they saw fit, following no particular guidance or structure.

#### Naluri Mobile App

**Figure 1:**
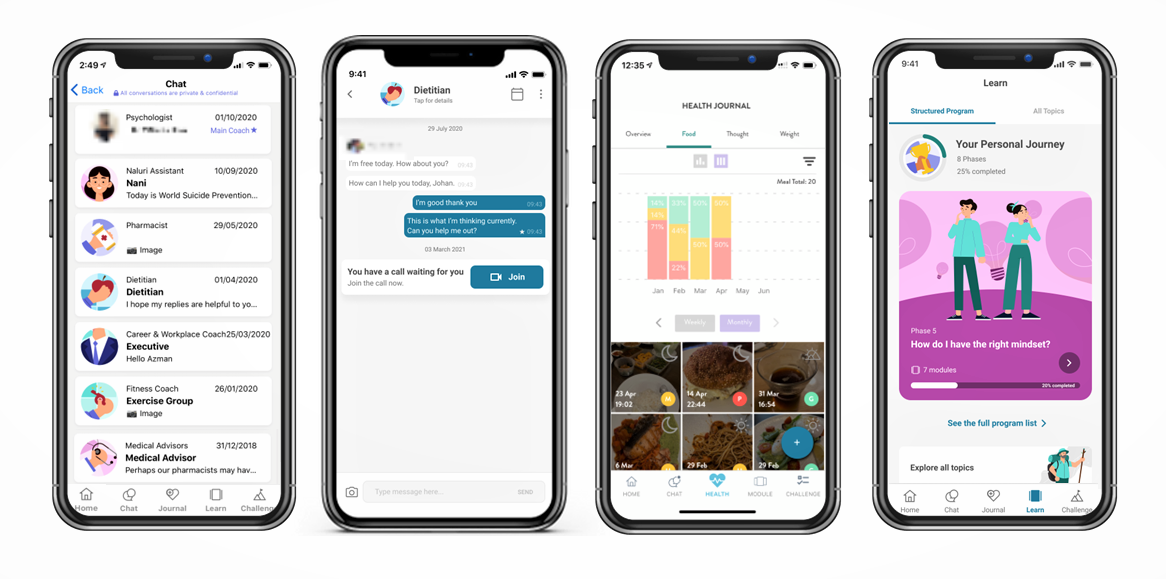
Naluri mobile app platform. (A) Chat home page with a multidisciplinary team of coaches. (B) Example of chat screen for text-based coaching with dietitians. (C) Health journal showing food journal uploads and overview. (D) Self-paced lessons and educational modules.

Both the Naluri CDMP and EAP are delivered digitally through the Naluri mobile app, a human-driven, artificial intelligence–augmented digital therapeutics platform that provides holistic digital care solutions aimed at predicting, preventing, and managing cardiometabolic and mental health conditions (Figure 1). In addition to a team of on-demand multidisciplinary coaches to provide accessible and on-demand coaching, the Naluri app is also optimized with evidence-based digital tools that facilitate behavioral change through features that promote goal setting and self-monitoring. These include habit trackers and artificial intelligence–augmented health journals that allow users to log and monitor their food intake, thoughts and emotions, weight, and BP; educational modules on topics such as resilience, diet and nutrition, managing chronic care, and exercise libraries; and the ability to set personalized challenges, as well as join support groups.

### Physical Outcomes

The physical health outcomes used in this study are sourced from secondary health data collected as part of an annual company-sponsored health screening for employees, which serves as the basis for this study’s outcome measures. In addition to physical health outcomes, participants’ self-reported ages and genders were also sourced from the data collected during the health screening.

We identify primary physical outcomes as changes in employee health profile, namely BMI, low-density lipoprotein (LDL) level, high-density lipoprotein (HDL) level, total cholesterol level, blood glucose level measured as HbA_1c_, systolic and diastolic BPs, body weight, and waistline circumference from 2021 to 2022. All physical health outcomes were measured by health practitioners specially contracted to conduct employee physical health screenings, which were then shared with Naluri for further evaluation.

### Statistical Analysis

All analyses were performed on RStudio version 2022.07.0+548, using R (version 4.2.1; R Core Team). Statistical tests were 2-sided and evaluated at a *P*<.05 significance threshold. We identify physical health outcomes at 2021 as “baseline” and physical health outcomes at 2022 as “follow-up.” Descriptive statistics were calculated for sociodemographic variables, such as age and gender, and for all outcome variables, reporting means and SDs for continuous data, and frequencies and percentages for categorical data. We report standardized mean differences to evaluate for incomparability across the 3 groups on age, gender, and all outcome variables at baseline, where standardized mean differences >0.1 would indicate evidence of imbalance across the 3 groups [32].

Linear mixed-effects models (LMMs) were used to assess the year-to-year changes in individual health outcomes, with time, group, and time by group interaction as fixed effects, and participants as random effects. LMMs, which allow for comparisons between- and within-groups at multiple time points, are considered to have more statistical power than traditional methods of evaluating interventions such as mixed ANOVAs or ordinary least squares regressions [33]. As group randomization was not possible for this study, participants were modelled as a random intercept to account for individual differences across all group conditions [34]. In addition, all models included gender, age, and baseline outcomes as covariates. Baseline covariate adjustments are a reliable and statistically efficient method of accounting for selection bias in nonrandomized observational studies [35], more so since any baseline differences across the 3 groups in this study are likely an inherent property of group membership [36]. All models were implemented through the *lme4* package [37] using restricted maximum likelihood estimation, and the significance of fixed effects was evaluated through *P* values derived using Satterthwaite approximations from omnibus ANOVA tests on the *lmerTest* package [38], which has been shown to produce lower type I error rates [39]. Post hoc pairwise comparisons were conducted to explore the direction of significant interactions between group and time using the *emmean*s package using the Tukey method to adjust for multiple comparisons [40]. Effect sizes were assessed using Cohen *d* statistics, which describes an effect size of 0.2 as small, 0.5 as medium, and 0.8 as large [41].

### Ethical Considerations

This study was not preregistered. This study was approved by the Medical and Research Ethics Committee, Ministry of Health Malaysia (NMRR ID-23-01212-PQH). Implied consent was obtained from participants when they accepted the Naluri mobile app privacy policy during the creation of their user accounts, which includes a clause stating that anonymized user data may be used for research purposes. The data used in this study was transferred and stored in compliance with HIPAA (Health Insurance Portability and Accountability Act) requirements. All raw data were anonymized and deidentified by independent parties before extraction for this study. No compensation was offered to participants as part of this study.

## Results

### Participant Characteristics

A total of 774 employees fulfilled the inclusion criteria and were included as participants in this study. Of the 300 invited to receive active coaching, 177 employees voluntarily participated in the CDMP. Of the remaining employees, 108 reported the use of the Naluri mobile app and received passive coaching, whereas 489 employees reported no use of Naluri’s digital services and thus received no coaching. The unequal sample sizes seen among the 3 groups reflect the real-world nature and voluntary enrollment of the employees into the offered digital health interventions.

The mean age of the overall sample was 38.51 (SD 8.32, range 21-81) years, and the majority of the participants identified as men (583/770, 75.71%). Table 1 presents the sample characteristics and baseline variables by group. Due to the nonrandomized nature of the study’s design, significant imbalances were evident for all baseline demographic and outcome variables between the 3 participant groups.

**Table 1:** Participant characteristics and baseline variable (N=774).

Variable			Active coaching (n=177)		Passive coaching (n=108)		No coaching (n=489)		Active vs passive coaching, SMD^a^			Active vs no coaching, SMD			Passive vs no coaching, SMD	
Age^b^ (years), mean (SD, range)			41.14 (7.78, 25-58)		35.80 (8.01, 22-52)		38.16 (8.33, 21-68)		0.68			0.37			–0.29	
**Gender^b^, n (%)**										0.75			0.13			0.61
	Women	29 (16.5)		53 (49.5)		105 (21.6)										
	Men	147 (83.5)		54 (50.5)		382 (78.4)										
Body weight (kg), mean (SD)			77.58 (10.63)		69.37 (11.40)		67.41 (11.20)		0.75			0.93			0.17	
BMI (kg/m^2^), mean (SD)			28.87 (3.58)		26.84 (3.67)		25.20 (3.65)		0.56			1.02			0.45	
HbA_1c_^c^ (%), mean (SD)			6.18 (1.56)		5.39 (0.61)		5.52 (0.88)		0.67			0.53			–0.17	
LDL^d^ (mg/dL), mean (SD)			148.34 (38.84)		117.39 (29.64)		125.59 (32.37)		0.90			0.64			–0.26	
HDL^e^ (mg/dL), mean (SD)			41.45 (8.74)		46.98 (12.17)		41.79 (9.90)		–0.52			–0.04			0.47	
Total cholesterol (mg/dL), mean (SD)			230.05 (38.53)		190.69 (34.05)		199.66 (36.56)		1.09			0.81			–0.25	
Systolic BP^f^, mean (SD)			123.22 (12.67)		112.32 (13.72)		115.41 (11.49)		0.83			0.65			–0.25	
Diastolic BP, mean (SD)			80.43 (6.60)		74.69 (6.87)		75.90 (6.93)		0.86			0.67			–0.18	
Waist circumference (cm), mean (SD)			96.32 (8.91)		89.59 (11.06)		87.57 (10.13)		0.67			0.92			0.19	

^a^SMD: standardized mean difference.

^b^For gender and age, total n=770 due to missing demographic values at baseline.

^c^HbA_1c_: hemoglobin A_1c_ or glycated hemoglobin.

^d^LDL: low-density lipoprotein.

^e^HDL: high-density lipoprotein.

^f^BP: blood pressure.

### Primary Health Outcomes

Table 2 presents descriptive statistics for all measured health outcomes at baseline and follow-up and omnibus results for fixed effects from the LMM analyses. As expected, the participants who were invited to receive active coaching reported poorer health outcomes in comparison to the other groups at baseline. Participants who received active coaching within the year reported improvements in their health outcomes at follow-up, with the most noticeable improvements being total cholesterol levels (mean difference [M_diff_]=–11.34 mg/dL). Those who engaged in passive coaching reported unchanging or slight deterioration in their health outcomes at follow-up, where slight improvements were only seen in HDL (M_diff_=+1.89 mg/dL) and total cholesterol (M_diff_=–0.78 mg/dL) levels. In comparison, participants who engaged in no coaching throughout the year recorded deteriorations in a majority of their health outcomes at follow-up, with the exception of improvements in HbA_1c_ (M_diff_=–0.03%) and HDL levels (M_diff_=+3.15 mg/dL). Results from LMMs showed a significant interaction effect of group×time for almost all health outcomes (*P*<.05) except for HDL (*P*=.09) and waist circumference (*P*=.06), indicating the presence of significant differences in the year-to-year change of health outcomes across the 3 groups.

**Table 2:** Means and SDs of health outcomes at baseline and follow-up by group with corresponding results of fixed effects from linear mixed models.

Variable and group			Baseline		Follow-up		Time							Group							Time×group			
			Mean (SD)		Mean (SD)		*F* test (*df*=1,1531)			*P* value			*F* test (*df*=2,1531)			*P* value			*F* test (*df*=2,1531)				*P* value	
**Body weight (kg)**								3.83			*.05* ^a^			9.02			<*.001*^a^			11.78				<*.001*^a^
	Active Coaching	77.58 (10.63)		77.01 (11.21)																				
	Passive Coaching	69.37 (11.40)		70.09 (11.79)																				
	No Coaching	67.41 (11.20)		67.96 (11.48)																				
**BMI (kg/m^2^)**								0.51			.47			3.36			*.04*			4.86				*.008* ^a^
	Active Coaching	28.87 (3.58)		28.67 (3.74)																				
	Passive Coaching	26.84 (3.67)		27.00 (3.82)																				
	No Coaching	25.20 (3.65)		25.38 (4)																				
**HbA_1c_^b^(%)**								4.87			*.03* ^a^			0.71			.49			3.68				*.03* ^a^
	Active Coaching	6.18 (1.56)		6.04 (1.30)																				
	Passive Coaching	5.39 (0.61)		5.39 (0.66)																				
	No Coaching	5.52 (0.88)		5.49 (0.80)																				
**LDL^c^ (mg/dL)**								36.51			<*.001*^a^			0.18			.84			6.86				*.001* ^a^
	Active Coaching	148.34 (38.84)		149.52 (39.38)																				
	Passive Coaching	117.39 (29.64)		126.61 (33.35)																				
	No Coaching	125.59 (32.37)		133.97 (32.23)																				
**HDL^d^ (mg/dL)**								64.07			<*.001*^a^			1.28			.28			2.32				*.01* ^a^
	Active Coaching	41.45 (8.74)		43.60 (8.06)																				
	Passive Coaching	46.98 (12.17)		48.87 (12.24)																				
	No Coaching	41.79 (9.90)		44.94 (10.46)																				
**Total cholesterol (mg/dL)**								12.39			<*.001*^a^			0.84			.43			11.97				<*.001*^a^
	Active Coaching	230.05 (38.53)		218.71 (41.94)																				
	Passive Coaching	190.69 (34.05)		189.81 (35.89)																				
	No Coaching	199.66 (36.56)		199.72 (35.48)																				
**Systolic BP^e^**								13.28			<*.001*^a^			1.06			.35			5.57				*.004* ^a^
	Active Coaching	123.22 (12.67)		122.73 (10.79)																				
	Passive Coaching	112.32 (13.72)		116.00 (13.63)																				
	No Coaching	115.41 (11.49)		117.34 (11.93)																				
**Diastolic BP**								0.13			.72			1.71			.18			5.18				*.006* ^a^
	Active Coaching	80.43 (6.60)		79.12 (5.16)																				
	Passive Coaching	74.69 (6.87)		75.81 (6.95)																				
	No Coaching	75.90 (6.93)		76.32 (6.85)																				
**Waist circumference (cm)**								1.62			.20			0.14			.87			2.76				.06
	Active Coaching	96.32 (8.91)		96.01 (8.52)																				
	Passive Coaching	89.59 (11.06)		89.92 (10.55)																				
	No Coaching	87.57 (10.13)		88.47 (10.49)																				

^a^Indicate statistical significance.

^b^HbA_1c_: hemoglobin A_1c_ or glycated hemoglobin.

^c^LDL: low-density lipoprotein.

^d^HDL: high-density lipoprotein.

^e^BP: blood pressure.

Tables 3 and 4 present results from post hoc pairwise comparison of estimated marginal means from the LMMs, reporting contrasts for within-group differences in health outcomes over time, and contrasts for between-group differences at follow-up measurement. Those who received active coaching reported significant improvements in HbA_1c_% (M_diff_=–0.14, *P*=.008), HDL (M_diff_=+2.14, *P*<.001), and total cholesterol levels (M_diff_=–11.45, *P*<.001) between the years of 2021 and 2022. While the Active Coaching group did record improving trends in body weight, BMI, systolic and diastolic BPs, and waist circumference, these improvements were not substantial enough to be considered statistically significant. In comparison, participants who engaged in passive coaching only reported a significant improvement in HDL levels (M_diff_=+1.95, *P*=.04) between the 2 time points, with the group recording deteriorations in a majority of their remaining outcomes, specifically in LDL levels (M_diff_=+9.10, *P*=.001) and systolic BP (M_diff_=+3.71, *P*=.005). Separately, employees who received no coaching at all between 2021 and 2022 also recorded larger deteriorations in year-to-year changes of health outcomes, with the No Coaching group recording significant deterioration in body weight (M_diff_=+0.56), *P*<.001), LDL levels (M_diff_=+8.47, *P*<.001), systolic BP (M_diff_=+1.93, *P*=.001), and waist circumference (M_diff_=+3.41, *P*=.009).

Pairwise contrasts of health outcomes at follow-up revealed significant between-group differences for body weight, BMI, LDL, and total cholesterol levels that are in favor of the Active Coaching group. Compared with the No Coaching group, those who received active coaching between 2021 and 2022 reported significantly lower body weight (*P*<.001), BMI (*P*=.001), LDL (*P*=.045), and total cholesterol (*P*<.001) at the follow-up screening. Similarly, the Active Coaching group also reported lower body weight than the Passive Coaching group (*P*<.001) at follow-up.

**Table 3:** Results of pairwise comparisons reporting estimated marginal means, mean differences, and SEs for within-group contrasts^a^.

Variable and group			Baseline		Follow-up		Within-group contrasts^b^						
			EMM^c^ (SE)		EMM (SE)		M_diff_^d^ (SE)		*t* test (*df*=767)		*P* value^e^		Cohen *d* (95% CI)
**Body weight (kg)**													
	Active Coaching	70.05 (0.16)		69.47 (0.16)		0.58 (0.21)		2.70		.08		0.29 (0.08 to 0.50)	
	Passive Coaching	69.93 (0.20)		70.67 (0.19)		–0.74 (0.27)		–2.67		.08		–0.37 (–0.64 to –0.10)	
	No Coaching	70.04 (0.10)		70.60 (0.10)		–0.56 (0.13)		–4.38		*<.001* ^f^		–0.28 (–0.41 to –0.16)	
**BMI (kg/m^2^)**													
	Active Coaching	26.31 (0.08)		26.10 (0.08)		0.21 (0.11)		1.90		.40		0.21 (–0.01 to 0.41)	
	Passive Coaching	26.25 (0.10)		26.41 (0.10)		–0.16 (0.14)		–1.14		.87		–0.16 (–0.42 to 0.11)	
	No Coaching	26.29 (0.05)		26.47 (0.05)		–0.18 (0.07)		–2.80		.06		–0.18 (–0.31 to –0.05)	
**HbA_1c_^g^ (%)**													
	Active Coaching	5.72 (0.03)		5.57 (0.03)		0.14 (0.04)		3.45		*.008* ^f^		0.37 (0.16 to 0.58)	
	Passive Coaching	5.61 (0.04)		5.62 (0.04)		–0.01 (0.05)		–0.12		>.99		–0.02 (–0.29 to 0.25)	
	No Coaching	5.63 (0.02)		5.61 (0.02)		0.02 (0.03)		0.89		.95		0.06 (–0.07 to 0.18)	
**LDL^h^ (mg/dL)**													
	Active Coaching	131 (1.32)		132 (1.32)		–1.09 (1.78)		–0.61		.99		–0.07 (–0.27 to 0.14)	
	Passive Coaching	128 (1.63)		137 (1.63)		–9.10 (2.29)		–3.98		*.001* ^f^		–0.54 (–0.81 to –0.28)	
	No Coaching	128 (0.83)		137 (0.83)		–8.47 (1.07)		–7.91		*<.001* ^f^		–0.51 (–0.63 to –0.38)	
**HDL^i^ (mg/dL)**													
	Active Coaching	42.80 (0.38)		44.90 (0.38)		–2.14 (0.52)		–4.12		*<.001* ^f^		–0.44 (–0.65 to –0.23)	
	Passive Coaching	43.0 (0.48)		44.90 (0.48)		–1.95 (0.67)		–2.93		*.04* ^f^		–0.40 (–0.67 to –0.13)	
	No Coaching	42.7 (0.24)		45.9 (0.24)		–3.17 (0.31)		–10.16		*<.001* ^f^		–0.65 (–0.78 to –0.53)	
**Total cholesterol (mg/dL)**													
	Active Coaching	208 (1.53)		197 (1.53)		11.45 (2.04)		5.61		*<.001* ^f^		0.60 (0.39 to 0.81)	
	Passive Coaching	203 (1.87)		202 (1.87)		1.10 (2.62)		0.42		>.99		0.06 (–0.21 to 0.33)	
	No Coaching	204 (0.95)		204 (0.95)		–0.09 (1.23)		–0.078		>.99		–0.005 (–0.13 to 0.12)	
**Systolic BP^j^**													
	Active Coaching	118 (0.60)		117 (0.60)		0.49 (0.81)		0.61		.99		0.06 (–0.14 to 0.27)	
	Passive Coaching	116 (0.75)		120 (0.75)		–3.71 (1.04)		–3.55		*.005* ^f^		–0.49 (–0.75 to –0.22)	
	No Coaching	116 (0.38)		118 (0.38)		–1.93 (0.49)		–3.94		*.001* ^f^		–0.25 (–0.38 to –0.13)	
**Diastolic BP**													
	Active Coaching	77.9 (0.37)		76.6 (0.37)		1.24 (0.51)		2.42		.15		0.26 (0.05 to 0.47)	
	Passive Coaching	76.2 (0.47)		77.3 (0.47)		–1.13 (0.66)		–1.72		.52		–0.24 (–0.50 to 0.03)	
	No Coaching	76.5 (0.22)		76.9 (0.22)		–0.42 (0.31)		–1.37		.74		–0.09 (–0.21 to 0.04)	
**Waist circumference (cm)**													
	Active Coaching	90.34 (0.33)		90.05 (0.33)		0.29 (0.45)		0.66		.99		0.07 (–0.14 to 0.28)	
	Passive Coaching	89.83 (0.41)		90.19 (0.41)		–0.36 (0.58)		–0.63		.99		–0.09 (–0.36 to 0.18)	
	No Coaching	89.61 (0.21)		90.53 (0.21)		–0.92 (0.27)		–3.41		*.009* ^f^		–0.22 (–0.34 to –0.09)	

^a^Results are averaged over the 2 levels of gender.

^b^Contrast: baseline – follow-up.

^c^EMM: estimated marginal means.

^d^M_diff_: mean difference.

^e^*P* values are adjusted using the Tukey method for comparing a family of 6 estimates.

^f^Indicate statistical significance.

^g^HbA_1c_: hemoglobin A_1c_ or glycated hemoglobin.

^h^LDL: low-density lipoprotein.

^i^HDL: high-density lipoprotein.

^j^BP: blood pressure.

**Table 4:** Results of pairwise comparisons reporting estimated marginal mean differences and SEs for between-group contrasts at follow-up.

Groups			Variables and between-group contrasts at follow-up^a^						
			M_diff_^b^ (SE)		*t* test (*df*)		*P* value^c^		Cohen *d* (95% CI)
**Body weight (kg)**									
	No Coaching – Active Coaching	1.13 (0.18)		6.16 (1522)		*<.001* ^d^		0.56 (0.39 to 0.74)	
	No Coaching – Passive Coaching	–0.07 (0.22)		–0.32 (1529)		>.99		–0.04 (–0.25 to 0.18)	
	Passive Coaching – Active Coaching	1.99 (0.25)		4.77 (1528)		*<.001* ^d^		0.60 (0.35 to 0.84)	
**BMI (kg/m^2^)**									
	No Coaching – Active Coaching	0.38 (0.09)		3.99 (1519)		*.001* ^d^		0.37 (0.19 to 0.55)	
	No Coaching – Passive Coaching	0.07 (0.11)		0.62 (1529)		.99		0.07 (–0.15 to 0.28)	
	Passive Coaching – Active Coaching	0.31 (0.13)		2.39 (1527)		.16		0.30 (0.05 to 0.55)	
**HbA_1c_^e^(%)**									
	No Coaching – Active Coaching	0.03 (0.04)		0.89 (1529)		.95		0.08 (–0.10 to 0.26)	
	No Coaching – Passive Coaching	–0.01 (0.04)		–0.35 (1530)		>.99		–0.04 (–0.25 to 0.17)	
	Passive Coaching – Active coaching	0.05 (0.05)		0.94 (1527)		.94		0.12 (–0.13 to 0.36)	
**LDL^f^ (mg/dL)**									
	No Coaching – Active Coaching	4.34 (1.50)		2.89 (1528)		*.045* ^d^		0.26 (0.08 to 0.44)	
	No Coaching – Passive Coaching	–0.09 (1.81)		–0.05 (1530)		>.99		–0.01 (–0.22 to 0.44)	
	Passive Coaching – Active Coaching	4.43 (2.11)		2.10 (1525)		.29		0.27 (0.02 to 0.51)	
**HDL^g^ (mg/dL)**									
	No Coaching – Active Coaching	0.98 (0.43)		2.27 (1531)		.21		0.20 (0.03 to 0.37)	
	No Coaching – Passive Coaching	0.93 (0.53)		1.77 (1530)		.49		0.19 (–0.02 to 0.40)	
	Passive Coaching – Active Coaching	0.05 (0.61)		0.07 (1529)		>.99		0.01 (–0.26 to 0.25)	
**Total cholesterol (mg/dL)**									
	No Coaching – Active Coaching	7.24 (1.74)		4.17 (1525)		*<.001* ^d^		0.38 (0.20 to 0.56)	
	No Coaching – Passive Coaching	1.73 (2.07)		0.84 (1530)		.96		0.09 (–0.12 to 0.30)	
	Passive Coaching – Active Coaching	5.51 (2.43)		2.26 (1523)		.21		0.29 (0.04 to 0.54)	
**Systolic BP^h^**									
	No Coaching – Active Coaching	0.67 (0.69)		0.98 (1528)		.93		0.09 (–0.09 to 0.26)	
	No Coaching – Passive Coaching	–1.57 (0.83)		–1.90 (1530)		.41		–0.21 (–0.42 to 0.007)	
	Passive Coaching – Active Coaching	2.24 (0.96)		2.33 (1527)		.18		0.29 (0.05 to 0.54)	
**Diastolic BP**									
	No Coaching – Active Coaching	0.26 (0.43)		0.60 (1529)		.99		0.05 (–0.12 to 0.23)	
	No Coaching – Passive Coaching	–0.43 (0.52)		–0.83 (1532)		.96		–0.09 (–0.30 to 0.12)	
	Passive Coaching – Active Coaching	0.68 (0.60)		1.14 (1529)		.87		0.14 (–0.10 to 0.39)	
**Waist circumference (cm)**									
	No Coaching – Active Coaching	0.49 (0.38)		1.27 (1522)		.80		0.12 (–0.06 to 0.29)	
	No Coaching – Passive Coaching	0.34 (0.46)		0.75 (1527)		.98		0.08 (–0.06 to 0.29)	
	Passive Coaching – Active Coaching	0.15 (0.53)		0.28 (1526)		>.99		0.03 (–0.21 to 0.28)	

^a^Results are averaged over the 2 levels of gender.

^b^M_diff_: mean difference.

^c^*P* values are adjusted using the Tukey method for comparing a family of 6 estimates.

^d^Indicate statistical significance.

^e^HbA_1c_: hemoglobin A_1c_ or glycated hemoglobin.

^f^LDL: low-density lipoprotein.

^g^HDL: high-density lipoprotein.

^h^BP: blood pressure.

## Discussion

### Principal Findings

In this observational study analyzing retrospective data of 774 employees, receiving active coaching by the Naluri digital CDMP was associated with statistically significant improvement in year-to-year changes in health outcomes. We found significant group×time interaction effects for body weight, BMI, HbA_1c_, LDL, total cholesterol, and systolic and diastolic BPs. Post hoc pairwise comparisons revealed that, of the 3 naturally occurring groups observed in this real-world study, those who engaged in passive coaching or no coaching at all recorded deteriorating trends in body weight, LDL, systolic BP, and waist circumference from 2021 to 202. Between-group contrasts also revealed that, at follow-up, employees who had received active coaching reported significant improvements in body weight, BMI, and total cholesterol compared with those who did not receive any form of coaching. Overall, our findings suggest that structured, intensive, and active digital health coaching was associated with an improvement in overall employee health status and minimizing chronic disease risk factors in an Indonesian workforce sample.

Our study adds to the growing evidence supporting the use of digital behavioral health coaching in improving health-related outcomes in the workplace. A systematic review of 22 randomized controlled trials found that digital health interventions in the workplace create a positive impact on health-related workplace outcomes such as diet, exercise, mental health, and job satisfaction [22]. Similarly, a systematic review of 22 studies found that digital health coaching was effective in managing and improving HbA_1c_, weight loss, and BMI [42]. Although the effect sizes observed in this study were primarily small to medium in magnitude, our findings are similar to that of Nkhoma et al [43], which similarly found a small effect (Hedges *g*=0.29) in a meta-analysis of digital interventions on HbA_1c_. Among studies looking into the Asian population, mobile-based digital workplace wellness interventions have been successful in reducing overall cardiovascular disease risk among hospital management workers in China [44], reducing the incidence of type 2 diabetes among male employees in India [45], and improving body weight, blood triglyceride, and systolic BP among university employees in Malaysia [46]. Although previous studies have demonstrated the effectiveness of workplace health promotion programs in improving employee metabolic health outcomes in Indonesia [47], to our knowledge, this study is the first to have looked into the real-world effects of workplace digital behavioral coaching among Indonesian employees.

Of the 3 groups of employees observed in our study, those who did not engage with or receive any form of digital coaching reported significantly poorer year-to-year health status compared with the other groups. Observational studies using propensity-matched controls have also demonstrated similar results, with Wilson et al [25] reporting year-to-year weight gain and an increase in fasting blood glucose in their propensity-matched control group. The trend in health outcomes among the participants in the No Coaching group offers a real-world glimpse of the year-to-year deterioration in health that can occur naturally and highlights the importance of preventative workplace health programs even for employees who are considered physically healthy. In a longitudinal study following a cohort of Singaporean employees, Sathish et al [48] observed a natural increase in physical inactivity, an increase in the prevalence of overweight or obesity, and significantly worsening dietary habits within 12 months. Similarly, large-scale studies have projected a 35.4% increase in diabetes prevalence by 2050 across the Southeast Asian region [49]. Given the intrinsic NCD health risks associated with traditional workplace environments [13], and the increasing stressors modern employees continue to face [50,51], our results reinforce the importance of workplace health interventions in reversing natural downward health trends and call attention to the health burdens that employers in the region will need to carry if no proactive action is taken.

Interestingly, despite having access to similar Naluri digital services as the Active Coaching group, we observed no change and even deterioration in a number of health outcomes among the group of employees who engaged in passive coaching. This may be attributed to the unstructured nature of the coaching received by this group of employees. Although limited evidence does suggest that unstructured and self-directed digital interventions can be effective in improving health outcomes [52,53], our findings are more aligned with the previous studies in this area that highlight the effectiveness of structured over unstructured digital interventions [54,55]. To this effect, our results highlight the strength of using Naluri’s digital tools within the context of a structured and theory-driven intervention framework as compared with passive, unstructured, and self-driven use. Separately, participant-related characteristics such as motivation and self-determination have been shown to be important drivers of behavioral change in digital interventions [56]. The mere knowledge that employees in this group were not at high risk during the baseline screening may have precluded them from developing the motivation and self-determination needed to improve their health [57], similar to what is seen in individuals at the precontemplation stage [31].

Currently, there is limited evidence that supports the use of digital health programs for improving physical outcomes in a Southeast Asian population [58]. To our knowledge, our findings are the first in the region to use real-world data, thus building the body of evidence supporting the use of digital health for real-world NCD prevention. Despite the relative infancy of digital health in Southeast Asia, the region is also known as one of the fastest adopters of mobile health technologies in the past decade [59], with Indonesians reporting one of the highest rates of smartphone health information-seeking behavior in the region [60]. To this effect, our findings highlight and reinforce the potential of digital health interventions as a cost-effective, resource-efficient, and scalable avenue for the prevention and management of NCDs in the region.

### Limitations

The findings of this study should be interpreted within the context of several limitations. All employees included in the study were given access to their company’s corporate-sponsored wellness initiatives within the period of 2021 to 2022, which were not limited to Naluri’s digital health coaching. The potential use of additional health improvement initiatives outside of those described in this study may have inflated or concealed within- or between-group differences in health outcomes at follow-up. In addition, given that the sample groups were retrospectively determined based on health screening data, self-selection, and natural usage patterns, outcomes of the study are likely susceptible to selection bias, despite our best efforts to control for this statistically. Admittedly, the nonrandomized and noncontrolled design of this study limits proper inferences of causality as well as limits the representativeness of our findings.

Furthermore, the retrospective and observational nature of this study precludes us from controlling for measured or unmeasured confounding variables such as mental health status or the existence of comorbid physical health conditions [61]. The omission of these potential confounding variables, though unavoidable in retrospective, observational studies [61], can in fact lead to an overestimation or an underestimation of the observed associations we see between coaching status and employee health outcome at follow-up. However, the observational nature of our study does offer a reflection on the real-world outcomes and implications of offering digital coaching and health improvement initiatives in the workplace. Separately, we did not look into participant engagement and adherence to the interventions, and this hinders any inferences on the dose-response relationship between the intervention and our study outcomes [62]. Despite these limitations, the real-world setting of our study provides ecological validity to our findings, prompting the need for more real-world studies of digital health use in the region, more so given the low adoption of digital interventions outside of randomized controlled trials [63]. To further contextualize the effectiveness of digital health interventions in the workplace, there is a need for future studies to look into the long-term outcomes and real-world cost-effectiveness of these programs.

### Conclusion

This study retrospectively investigated the year-to-year changes in employee health outcomes associated with a workplace digital behavioral coaching intervention. We found that an active coaching CDMP led to significant year-to-year improvements in multiple measures of employee health outcomes compared with those receiving passive coaching or no coaching at all. Despite methodological limitations, our study provides real-world evidence to support the use of digital workplace interventions in NCD prevention in the region.

## Abbreviations

BP: blood pressure
CDMP: chronic disease management program
EAP: employee assistance program
HbA1c: hemoglobin A1c
HDL: high-density lipoprotein
HIPAA: Health Insurance Portability and Accountability Act
LDL: low-density lipoprotein
LMM: linear mixed-effects model
Mdiff: mean difference
NCD: noncommunicable disease
WHO: World Health Organization

## Data Availability

The data used and analyzed for this study are available from the corresponding author on reasonable request.
